# Estimating body mass of wild pigs (*Sus scrofa*) using body morphometrics

**DOI:** 10.1002/ece3.9853

**Published:** 2023-03-08

**Authors:** Carolina Baruzzi, Nathan P. Snow, Kurt C. Vercauteren, Bronson K. Strickland, Jacques S. Arnoult, Justin W. Fischer, Michael P. Glow, Michael J. Lavelle, Benjamin A. Smith, Daryl Steakley, Marcus A. Lashley

**Affiliations:** ^1^ Department of Wildlife Ecology and Conservation, North Florida Research and Education Center University of Florida Quincy Florida USA; ^2^ Department of Wildlife, Fisheries, and Aquaculture Mississippi State University Mississippi State Mississippi USA; ^3^ National Wildlife Research Center USDA/APHIS Fort Collins Colorado USA; ^4^ Department of Wildlife Ecology and Conservation University of Florida Gainesville Florida USA

**Keywords:** body weight, morphometric characteristics, physical condition, population management, *Sus scrofa*, wild pig, wildlife management

## Abstract

Wild pigs (*Sus scrofa*) are invading many areas globally and impacting biodiversity and economies in their non‐native range. Thus, wild pigs are often targeted for eradication efforts. Age‐ and sex‐specific body measurements are important for informing these eradication efforts because they reflect body condition, resource availability, and fecundity, which are common indicators of population trajectory. However, body mass is often difficult to collect, especially on large individuals that require specialized equipment or multiple people to weigh. Measurements that can be rapidly taken by a single land or wildlife manager on any size wild pig without aid from specialized equipment would be beneficial if they accurately infer wild pig body mass. Our goals were to assess whether morphometric measurements could accurately predict wild pig body mass, and to provide tools to directly input these measures and estimate wild pig body mass. Using linear models, we quantified the relationship between body mass and morphometric measurements (i.e., body length, chest girth, ear length, eye to snout length, hindfoot length, shoulder length, and tail length) from a subset (*n* = 102) of wild pigs culled at the Mississippi Alluvial Valley, Mississippi, USA. We evaluated separate models for each individual morphometric measurement. We then used the model coefficients to develop equations to predict wild pig body mass. We validated these equations predicting body mass of 1592 individuals collected across eight areas in Australia, Guam, and the USA for cross‐validation. Each developed equation remained accurate when cross‐validated across regions. Body length, chest girth, and shoulder length were the morphometrics that best predicted wild pig body mass. Our analyses indicated it is possible to use the presented equations to infer wild pig body mass from simple metrics.

## Graphical abstract

We developed a set of equations to predict wild pig body mass from other morphometric measures. Data were collected and validated from eight different areas in the United States, Australia, and Guam.

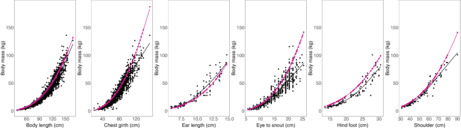

## 1 INTRODUCTION

Wild pigs (i.e., individuals of the *Sus scrofa* species whose population are the result of genetic mixing of wild boar and domestic pigs; Keiter et al., 2016) are considered one of the hundred world's most invasive species and their populations are rapidly expanding (Lowe et al., 2000). For example, in the United States, wild pigs have colonized at least 35 states and continue to expand their range (Lewis et al. 2019; McKee et al., 2020). Their expansion into non‐native ranges has resulted in widespread economic losses and negatively impacted flora and fauna (Barrios‐Garcia & Ballari, 2012; Ivey et al., 2019; McDonough et al., 2022; McKee et al., 2020; Pimentel, 2007). Because of their economic and ecological impacts, wild pigs are frequently the target of eradication efforts (e.g., trapping, aerial gunning, ground shooting; Gaskamp et al., 2021; Massei et al., 2011; McKee et al., 2020), but several factors such as high reproductive rates, general dietary habits, and dispersal capability make this species extremely challenging to eradicate (Ditchkoff et al., 2017).

Sustained monitoring of wild pig populations is important to measure the success of eradication or control efforts (Massei et al., 2011). However, population control is not typically based on knowledge of population densities, likely because accurately monitoring population dynamics is difficult (Davis et al., 2020; Engeman et al., 2013; Guerrasio et al., 2022), and managing such a fast‐growing species requires constant, rapid management responses. Morellet et al. (2007) suggested that indicators of ecological change (e.g., body mass or female reproductive success) can infer population dynamics and species relationships with available resources, and ultimately help inform population control efforts. Our understanding of wild pig population dynamics is still limited (Beasley et al., 2018), and development of new tools for population monitoring, such as these indicators of ecological change, should be useful in eradication efforts.

Wild pig morphometric measurements that are easy to measure may help facilitate monitoring the status wild pig populations, if they provide a practical means to estimate important population parameters. Animal morphometric measurements are often used to estimate population vital rates, as they often can be indicative of the relationship between a species and its environment (e.g., Castellano et al., 2000; Hossain et al., 2019; Su et al., 2018; Vannini et al., 2021). Body mass of animals is one of the most used morphometric measurements to estimate population vital rates as it may be indicative of the relationship between population density and resource availability (e.g., Bjorndal et al., 2000; Delahay et al., 2006; Lashley et al., 2015). Body mass has been used as an index of fecundity and survival in a number of ungulate species such as white‐tailed deer (*Odocoileus virginianus*; Strickland et al., 2008), red deer (*Cervus elaphus*; Albon et al., 1983), caribou (*Rangifer tarandus*; Cameron 1993), and ibex (*Capra ibex*; Bassano et al., 2003). In the case of wild pigs, monitoring body mass should provide a useful indicator of their reproductive potential (Bieber & Ruf, 2005; Dexter, 2003). This information could aid eradication efforts to efficiently allocate resources, monitor success, and plan future management strategies. Moreover, body mass may be indicative of environmental conditions and serve as an indicator of which age classes are most vulnerable to culling. For example, Bieber and Ruf (2005) suggested that population control of wild pigs would be most effective if targeting adult females when environmental conditions are limiting, and juveniles when environmental conditions are good. Body mass has also been shown to be indicative of general body condition in wild pigs (Risco et al., 2018), which is likely a more accurate and quantifiable measure of the relationships between wild pig populations and their resources than environmental conditions per se. For these reasons, wild pig body mass has been recently identified as one of the parameters to report to promote comparable monitoring programs and management (Pascual‐Rico et al., 2022). Thus, body mass should provide managers with fundamental information that could maximize inference gained from wild pig control efforts.

Body mass can be difficult to obtain based on equipment limitations, accessibility, time restrictions, and size of the target species (Bassano et al., 2003; Talbot & McCulloch, 1965). Thus, it would be beneficial if body mass could be inferred from more easily obtainable measurements such as chest girth or body length (e.g., Cam et al., 2010; Risco et al., 2018). Relating easily measurable morphometric measurements to body mass could yield to quickly and reliably gain information on the reproductive potential of a wild pig population and inform management actions as shown in other ungulate species (e.g., Bartareau, 2019; Cook et al., 2003; Millspaugh & Brundige, 1996; Rideout & Worthen, 1975; Wallin et al., 1996). As such, the goal of our study was to develop tools to estimate wild pig body mass from morphometric measures across different regions and sexes. Because the use of remote sensing is becoming prevalent in wildlife management, we aimed at developing a gender‐neutral tool to be potentially used with remotely collected data (Smith et al., 2021). We collected morphometric measurements in eight areas around Australia, Guam, and the USA. We quantified the relationship between body morphometric measurements that could be easily collected and body mass of wild pigs.

## 2 METHODS

### 2.1 Study regions

We initiated this study in Mississippi, USA, and successively expanded it for validation purposes. In Mississippi, data were collected in two regions (i.e., Mississippi Alluvial Valley and the Mississippi Interior Flatwoods) between 2017 and 2018 (see Supplementary Information 3 for region‐specific sample size per year). We collected morphometric data from wild pigs across three counties in the Mississippi Alluvial Valley (i.e., Leflore, Tallahatchie, Sunflower), and one county in the Mississippi Interior Flatwoods (i.e., properties in Clay County). Morphometric measures were also collected from two distinct regions in Texas between 2016 and 2021: Camp Bullis Military Training Reservation, Bexar County, and two counties in northcentral Texas (i.e., Motley and Wilbarger counties). In the other study areas, data were collected in one region per area. Specifically, wild pigs were trapped in: Macon County, Alabama in 2019 and 2021; St. George, Queensland, Australia, in 2018; northern Guam in 2021; and Hakalau National Wildlife Refuge, Hawaii, in 2021 and 2022.

### 2.2 Morphometric measure collection

Pigs were captured using various traps baited with corn. Once wild pigs were captured, they were then euthanized (IACUC Protocol No. 14–100 for Mississippi samples). Measurements collected by USDA personnel were done conducted by following several USDA IACUC‐approved studies (Sanders et al., 2020; Snow et al., 2019; Snow et al., 2022; Snow, Halseth, et al., 2021; Snow & VerCauteren, 2019; Snow, Wishart, et al., 2021). Body mass and other morphometric data were gathered postmortem. Body mass was measured to the nearest kg. In Mississippi, we recorded morphometric measures for: body length, chest girth, ear length, eye to snout length, hindfoot length, shoulder length, and tail length (see Table 1 for measurement collection details). Body mass, body length, and chest girth were recorded in every area outside Mississippi, while eye to snout length was recorded only in Alabama, Hawaii, and South Texas. Body mass, body length, and eye to snout length were recorded as in Mississippi. Chest girth was recorded by measuring the widest length from the center of sternum behind the scapula to the spine and multiplying this value by 2. Although we expect our measured individuals to have mild body asymmetries, bilateral symmetry is a fundamental characteristic of vertebrate body plans, so we did not expect this chest girth measurement to affect our inference. Hindfoot length and tail length were not measured outside Mississippi. Because shoulder length was measured as the shorter distance between the spine to the bottom of the hoof outside Mississippi (i.e., vs. from the tip of the scapula to the hoof), we decided not to use this measure for individuals outside Mississippi.

**TABLE 1:** Collection methods of morphometric measurements for wild pigs measured in Mississippi.

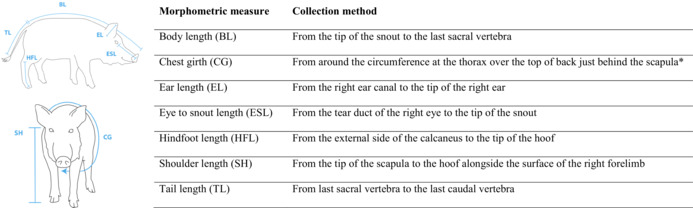

In all other areas (i.e., Alabama, Australia, Guam, Hawaii, and Texas), body length and eye to snout length were measured as in Mississippi. *Indicates that measure chest girth was recorded differently outside Mississippi. Specifically, it was recorded by measuring from the center of sternum to the spine and multiplying this value by 2. We used hindfoot, tail, and shoulder length only from individuals measured in Mississippi.

### 2.3 Analysis

We first identified the morphometric measurements (Table 1) that could produce accurate estimates of wild pig body mass by analyzing the relationships of each measurement in separate models (i.e., one measure per model) for the entire dataset, each study area, and for different sexes in each study area. We tested the relationship between each morphometric measure and body mass for the entire dataset and subsets of the data because we wanted to find which morphometric measures could most reliably predict wild pig body mass across different regions and sexes. We analyzed an individual morphometric measure per model for two reasons: first, morphometric measures were highly correlated (Supplementary Information 1 and 2), which would generate multicollinearity issues if multiple independent variables would be tested in the same model. Second, we aimed to provide an easy and quick method to assess wild pig body mass, and using multiple measures may discourage managers from collecting measurements. Although morphometric measures are generally easy to collect, field work related to wild pig management is intensive and full of tasks. Adding the task of collecting multiple morphometric measures could still be challenging and time‐consuming in this context, especially if several individuals are measured.

In each model, both body mass and morphometric measures were log transformed to meet linearity assumptions. We used coefficient of determination (*R* ^2^) as a means of determining the proportion of the variation in wild pig body mass that was explained by each morphometric measure. We decided not to develop equations for metrics that explained little variation in the data as we expected them not to produce reliable estimates. We considered a morphometric measure as an acceptable candidate to build and test our equations if it explained at least 90% of the variation in the body mass data in half of the analyses. Using the morphometric measures we considered acceptable, we built an equation per morphometric measure to estimate wild pig body mass. Specifically, we used coefficients from the Mississippi Alluvial Valley models and compared estimates of wild pig body mass with morphometric measures collected in other study areas by plotting these values. We also calculated Spearman's rank correlation coefficients and average percent difference between measured and estimated body mass. We used the Spearman's rank correlation because data were non‐normally distributed. All data were analyzed in Program R (Version 3.6.0; R Core Team, 2019).

## 3 RESULTS

We measured 1656 wild pigs over the course of the study (see Supplementary Information 3 for sample size in each region by year and sex). Morphometric measures across regions appeared to have a consistent relationship with body mass (Supplementary Information 4). When regressing body mass over morphometric measures collected across all study regions, the morphometric measures tested explained more than 90% of the variation in body mass (*R* ^*2*^_body length_ = 0.96; *R* ^*2*^_chest girth_ = 0.93; *R* ^2^_eye to snout length_ = 0.92) and the model that better fit our data included body length as independent variable (AIC_body length_ = −1259.76; AIC_chest girth_ = −115.89; AIC_eye to snout length_ = 128.64). Among the morphometric measures collected only in Mississippi, ear, hindfoot, and shoulder length (*R* ^*2*^_ear length_ = 0.92; *R* ^*2*^_hindfoot length_ = 0.91, *R* ^*2*^_shoulder length_ = 0.96) explained more than 90% of the variation in body mass while tail length explained almost 80% (*R* ^*2*^_tail length_ = 0.79). Among these measures, the model that better fit our data included shoulder length as independent variable (AIC_ear length_ = 38.64; AIC_hindfoot length_ = 62.54, AIC_shoulder length_ = −81.68, AIC_tail length_ = 221.31).

When regressing body mass over the morphometric measures collected in different study areas (Supplementary Information 5A) and sexes within each study areas (Supplementary Information 5B,C), the only morphometric measure that consistently explained less than 90% of the variation in body mass was tail length. Across all areas, morphometric measures collected in Guam were the only measures that consistently explained less than 80% of the variation in body mass (Supplementary Information 5).

**FIGURE 1:**
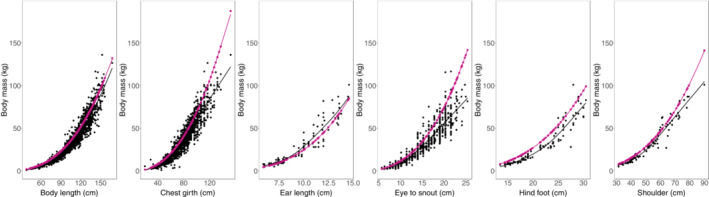
Relationship between body length, chest girth, ear length, eye to snout length, hind foot length, or shoulder length and estimated (pink lines and points) and measured (black lines and points) body mass. Ear length and hind foot were collected only in the Mississippi Interior Flatwoods. The shoulder length panel represents data from only Mississippi Interior Flatwoods because in the other regions, shoulder length was measured differently than in Mississippi Alluvial.

We used all morphometric measures but tail length to build six equations that can be used to estimate wild pig body mass in the field. All estimates were highly correlated to the measured wild pig body mass (Figure 1 and Table 2). However, body length, chest girth, and shoulder length appeared to be more precise when visually assessing plots (Figure 1) and comparing measured and estimated body mass (Table 2). The other measures appeared to be less precise, especially when body mass increased (Figure 1). This was also reflected when calculating the average percent difference between measured and estimated body mass for eye to snout and hindfoot length, but not ear length (Table 2). As such, we provide an Excel file and an R Shiny app (i.e., https://kof3q9‐carolinabaruzzi.shinyapps.io/WildPigMorphometric/) that can be used to directly input each of the morphometric measures considered (i.e., body length, chest girth, and shoulder length) to predict body mass with our equations (Supplementary Information 6).

**TABLE 2:** Equation formula used to estimate wild pig body mass from each morphometric measure and relative results.

Measure	Formula	*r*_s_	Mean difference (%)	Mean difference (kg) ± SE	Mean absolute error (kg)	Root mean squared error (kg)
Body length	BM = e^−9.56 + 2.82 * ln(BL)^	0.98	15.9	4.08 ± 0.17	5.64	7.73
Chest girth	BM = e^−6.73 + 2.38 * ln(CG)^	0.96	19.5	5.83 ± 0.22	7.32	10.46
Ear length	BM = e^−4.3 + 3.26 * ln(EL)^	0.95	17.8	3.32 ± 0.87	6	9.18
Eye to snout length	BM = e^−3.78 + 2.7 * ln(ESL)^	0.96	25.1	9.03 ± 0.47	10.59	15.46
Hindfoot length	BM = e^−5.94 + 3.08 * ln(HFL)^	0.97	26	8.74 ± 0.89	9.63	12.4
Shoulder length	BM = e^−7.38 + 2.74 * ln(SH)^	0.98	15.6	4.4 ± 0.7	5.42	8.17

## 4 DISCUSSION

We found that morphometric measurements acceptably predicted body mass in both male and female wild pigs. The ability to readily estimate body mass from simple morphometric measures of culled individuals is a valuable tool that can be used by wildlife or land managers to inform eradication efforts (Bieber & Ruf, 2005). As the morphometric measures in this study are easy to collect, they could generate a substantial amount of data to monitor wild pig body mass in different regions.

In our study, body length, chest girth, and shoulder length were the measures that most accurately predicted wild pig body mass. Among these measures, shoulder length was the only one validated using data collected only in Mississippi. For this reason, we recommend interpreting results from shoulder length estimates with caution, and pairing them with other estimates obtained from body length and chest girth if possible. Although chest girth can be hard to measure on large individuals, our body mass predictions were still accurate when using chest girth collected measuring half of the chest girth length (i.e., from the center of the sternum to the spine), which could promote measurement collection. However, we still recommend measuring the full chest girth, when possible, as some individuals may exhibit body asymmetries that may make body mass predictions less accurate.

Although we cross validated our estimates and our equations appeared to be able to estimate wild pig body mass, regional resource, and genetic variation may generate differences among morphometric measures. For example, white‐tailed deer (*Odocoileus virginianus*) morphometric measurements have been linked to regional variation in resource availability in Mississippi (Jones et al., 2010; Michel et al., 2016). Seasonal or local resource fluctuation could also affect wild pig body mass, and chest girth measures would likely be the most sensitive to these fluctuations. Although low regional resource availability should limit wild pig growth and some morphometric measures (e.g., body length and shoulder length), abrupt local resource fluctuations are likely to affect body condition and fat storage, which could cause changes in chest girth (Risco et al., 2018). As such, chest girth may be the morphometric measure that most accurately predicts wild pig body mass when local resources are fluctuating. However, body mass largely reflects individual physiological state, so we do not expect other morphometric measures to represent body mass less accurately unless resource fluctuations are extreme (Smiley et al., 2022).

The ability to establish a link between wild pig body mass and morphometric measures to monitor variations in regional or local resource availability is useful for managers as wild pig physiology and survival appear to be particularly sensitive to resource availability (Bieber & Ruf, 2005; Gamelon et al., 2017; Toïgo et al., 2008). Although our equations performed well when estimating wild pig body mass from different data sources, it should be noted that the individuals collected in our study come from *S. scrofa* invasive range. For this reason, results should be interpreted with caution if estimating pure Eurasian wild boar body mass. Moreover, a heritable component of body morphometrics has been demonstrated in several ungulate species (Herfindal et al., 2014; Kruuk et al., 2000; Rönnegård & Danell, 2003), including across *Sus scrofa* subspecies (e.g., Apollonio et al., 1988). Small, isolated populations maybe more affected by individual variation. In our study, morphometric measures collected in Guam explained less of the variation in body mass compared to other areas, which may be due to a founder effect, or a reduced sample size compared to other areas. Thus, variation in the accuracy of wild pig body mass estimation may vary across regions. Overall, our study confirmed the validity of using morphometric measurements to infer wild pig body mass and managers could efficiently estimate it to inform their management decisions (e.g., individuals to be targeted during culling) by monitoring population status (Bieber & Ruf, 2005).

## AUTHOR CONTRIBUTIONS

**Carolina Baruzzi:** Formal analysis (lead); investigation (lead); methodology (equal); visualization (lead); writing – original draft (lead); writing – review and editing (lead). **Nathan P. Snow:** Data curation (equal); investigation (equal); methodology (equal); writing – original draft (equal); writing – review and editing (equal). **Kurt C. Vercauteren:** Data curation (equal); funding acquisition (equal); investigation (equal); resources (equal); writing – original draft (equal); writing – review and editing (equal). **Bronson K. Strickland:** Conceptualization (equal); funding acquisition (equal); investigation (equal); methodology (equal); project administration (equal); resources (equal); writing – original draft (equal); writing – review and editing (equal). **Jacques S. Arnoult:** Data curation (equal); writing – review and editing (equal). **Justin W. Fischer:** Data curation (equal); project administration (equal); writing – review and editing (equal). **Michael P. Glow:** Data curation (equal); writing – review and editing (equal). **Michael J. Lavelle:** Data curation (equal); writing – review and editing (equal). **Benjamin A. Smith:** Data curation (equal); writing – review and editing (equal). **Daryl Steakley:** Data curation (equal); writing – review and editing (equal). **Marcus A. Lashley:** Conceptualization (equal); formal analysis (equal); funding acquisition (equal); investigation (equal); methodology (equal); resources (equal); visualization (equal); writing – original draft (equal); writing – review and editing (equal).

## Supporting information


Appendix S1

Appendix S2

## Data Availability

The data used to build the morphometric equations on GitHub at https://github.com/CarolinaBaruzzi/WildPigMorphometrics.git.
