# Supplementary material for: Estimating body mass of wild pigs (Sus scrofa) using body morphometrics
Source: Ecol Evol. 2023 Mar 8;13(3):e9853. doi: 10.1002/ece3.9853 (PMC9994471; doi:10.1002/ece3.9853)
Supplement: Supplementary file 1 — Appendix S1 [file ECE3-13-e9853-s001.docx]

**Supplementary Information 1.** Spearman's rank correlation coefficients between pairs of morphometric measures across all our study areas. Asterisks indicate significance (i.e., *** < 0.001, ** > 0.001 and < 0.01, * > 0.01 and < 0.05). Plot was performed (including coefficients and p-values) using the PerformanceAnalytics package in R (Peterson and Carl 2019).

**Supplementary Information 2.** Spearman's rank correlation coefficients between pairs of morphometric measures collected in Mississippi (i.e., Mississippi Alluvial Valley and Mississippi Interior Flatwoods), USA. Tail length, hindfoot length, and ear length were collected only in these areas. Asterisks indicate significance (i.e., *** < 0.001, ** > 0.001 and < 0.01, * > 0.01 and < 0.05). Plot was performed (including coefficients and p-values) using the PerformanceAnalytics package in R (Peterson and Carl 2019).

**Supplementary Information 3.** Number of individuals sampled in each region by year and sex. NA indicates the sex of an individual has not been recorded.

| **Region** | **Year** | **Sex** | **n** |
| --- | --- | --- | --- |
| Alabama | 2019 | Female | 16 |
| Alabama | 2019 | Male | 19 |
| Alabama | 2021 | Female | 59 |
| Alabama | 2021 | Male | 33 |
| Alabama | 2021 | NA | 1 |
| Australia | 2018 | Female | 39 |
| Australia | 2018 | Male | 24 |
| Guam | 2021 | Female | 22 |
| Guam | 2021 | Male | 22 |
| Hawaii | 2021 | Female | 120 |
| Hawaii | 2021 | Male | 112 |
| Hawaii | 2021 | NA | 1 |
| Hawaii | 2022 | Female | 119 |
| Hawaii | 2022 | Male | 128 |
| Hawaii | 2022 | NA | 1 |
| Mississippi Alluvial Valley | 2017 | Female | 22 |
| Mississippi Alluvial Valley | 2017 | Male | 25 |
| Mississippi Alluvial Valley | 2018 | Female | 24 |
| Mississippi Alluvial Valley | 2018 | Male | 31 |
| Mississippi Interior Flatwoods | 2018 | Female | 45 |
| Mississippi Interior Flatwoods | 2018 | Male | 53 |
| North Texas | 2018 | Female | 56 |
| North Texas | 2018 | Male | 63 |
| North Texas | 2018 | NA | 6 |
| North Texas | 2020 | Female | 81 |
| North Texas | 2020 | Male | 69 |
| North Texas | 2020 | NA | 2 |
| North Texas | 2021 | Female | 54 |
| North Texas | 2021 | Male | 33 |
| South Texas | 2016 | Female | 12 |
| South Texas | 2016 | Male | 7 |
| South Texas | 2017 | Female | 215 |
| South Texas | 2017 | Male | 142 |


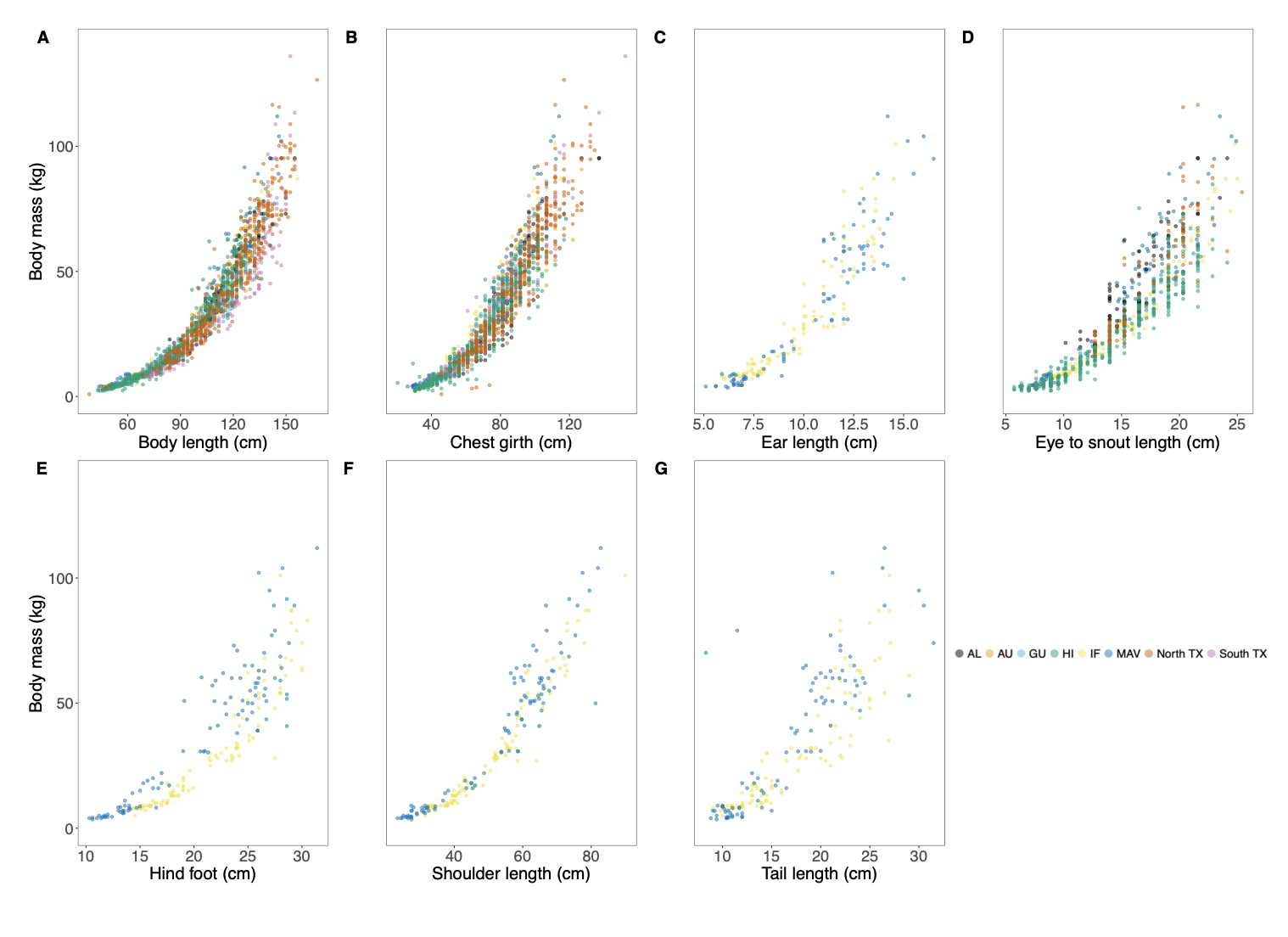
**Supplementary Information 4.** Relationship between body mass and morphometric measures across regions. AL is the abbreviation for Alabama, AU for Australia, GU for Guam, HI for Hawaii, IF for Mississippi Interior Flatwoods, MAV for Mississippi Alluvial Valley, North TX for North Texas, and South TX for South Texas.

**Supplementary Information 5.** Coefficient of determination for each morphometric measure in different regions A) irrespective of sex, B) for female wild pigs, and C) for male wild pigs. Missing cells indicate that specific morphometric measures have not been collected in a region.

| **A)** | **Body length** | **Chest girth** | **Ear length** | **Eye to snout length** | **Hind foot length** | **Shoulder length** | **Tail length** |
| --- | --- | --- | --- | --- | --- | --- | --- |
| **Alabama** | 0.92 | 0.84 |  | 0.76 |  |  |  |
| **Australia** | 0.96 | 0.89 |  |  |  |  |  |
| **Guam** | 0.67 | 0.78 |  |  |  |  |  |
| **Hawaii** | 0.98 | 0.94 |  | 0.94 |  |  |  |
| **Mississippi Alluvial Valley** | 0.97 | 0.97 | 0.93 | 0.96 | 0.93 | 0.96 | 0.75 |
| **Mississippi Interior Flatwoods** | 0.97 | 0.97 | 0.91 | 0.98 | 0.95 | 0.96 | 0.87 |
| **North Texas** | 0.96 | 0.95 |  |  |  |  |  |
| **South Texas** | 0.95 | 0.84 |  | 0.83 |  |  |  |
| **B)** |  |  |  |  |  |  |  |
| **Alabama** | 0.91 | 0.82 |  | 0.78 |  |  |  |
| **Australia** | 0.94 | 0.85 |  |  |  |  |  |
| **Guam** | 0.63 | 0.78 |  |  |  |  |  |
| **Hawaii** | 0.97 | 0.94 |  | 0.94 |  |  |  |
| **Mississippi alluvial valley** | 0.97 | 0.96 | 0.93 | 0.96 | 0.92 | 0.95 | 0.79 |
| **Mississippi interior flatwoods** | 0.97 | 0.96 | 0.87 | 0.98 | 0.93 | 0.94 | 0.86 |
| **North Texas** | 0.96 | 0.94 |  |  |  |  |  |
| **South Texas** | 0.95 | 0.87 |  | 0.79 |  |  |  |
| **C)** |  |  |  |  |  |  |  |
| **Alabama** | 0.91 | 0.82 |  | 0.78 |  |  |  |
| **Australia** | 0.94 | 0.85 |  |  |  |  |  |
| **Guam** | 0.62 | 0.78 |  |  |  |  |  |
| **Hawaii** | 0.97 | 0.94 |  | 0.94 |  |  |  |
| **Mississippi alluvial valley** | 0.98 | 0.98 | 0.93 | 0.97 | 0.95 | 0.97 | 0.73 |
| **Mississippi interior flatwoods** | 0.98 | 0.97 | 0.94 | 0.98 | 0.97 | 0.97 | 0.89 |
| **North Texas** | 0.96 | 0.94 |  |  |  |  |  |
| **South Texas** | 0.95 | 0.87 |  | 0.79 |  |  |  |

**References**

Peterson BG, Carl P (2019). PerformanceAnalytics: Econometric Tools for Performance and Risk Analysis. R package version 1.5.3. https://CRAN.R project.org/package=PerformanceAnalytics
